# Multimodal MRI radiomics-clinical fusion model predicts intravenous glucocorticoid response in thyroid eye disease

**DOI:** 10.3389/fendo.2025.1726947

**Published:** 2026-01-20

**Authors:** Yanhu Zhou, Fei Jia, Xuelian Zhao, Xiaojin Ma, Tao Chang, Shunyu Yao, Kuanyu Che, Jing Zhang

**Affiliations:** 1 The Second Hospital & Clinical Medical School, Lanzhou University, Lanzhou, China; 2 Department of Imaging, The First People’s Hospital of Lanzhou City, Lanzhou, China; 3 Gansu University of Chinese Medicine, Lanzhou, China; 4 Gansu Medical MRI Equipment Application Industry Technology Center, Lanzhou, China

**Keywords:** intravenous glucocorticoids, multimodal magnetic resonance imaging, nomogram, prediction mode, radiomics, thyroid eye disease, treatment response

## Abstract

**Background:**

This study aimed to develop a multimodal MRI radiomics-clinical fusion model for predicting intravenous glucocorticoid (IVGC) treatment response in patients with thyroid eye disease (TED).

**Methods:**

In this retrospective multicenter study, 108 TED patients (78 responders, 30 non-responders) from two institutions (January 2020–December 2024) were included, and treatment response was assessed at 12 weeks after completion of therapy. Patients were randomly split into training and test sets (8:2). All patients received a standardized intravenous methylprednisolone regimen (total dose 4.5 g over 12 weeks) according to EUGOGO recommendations. Univariate logistic regression was used to identify clinical predictors associated with response. Radiomics features and deep transfer learning (DTL) features were extracted from pretreatment T1-weighted imaging (T1WI) and fat-suppressed T2-weighted imaging (T2WI-FS). Feature selection followed a three-step pipeline (t-test, Pearson correlation filtering, and LASSO with 10-fold cross-validation), and a radiomics–deep learning fused (RDL) model was built. A combined model integrating the RDL score with independent clinical predictors was constructed and visualized as a nomogram. Model performance was evaluated using ROC/AUC, calibration curves, and decision curve analysis (DCA), and AUCs were compared using the DeLong test.

**Results:**

Disease duration and Clinical Activity Score (CAS) were independent predictors of IVGC response (P < 0.05). The RDL model outperformed radiomics-only models, achieving AUCs of 0.894 (95% CI: 0.804–0.984) in the training set and 0.804 (95% CI: 0.595–1.000) in the test set. The combined model demonstrated further improved performance, with training and test set AUCs of 0.916 (0.837–0.994) and 0.862 (0.702–1.000), respectively, along with better calibration and higher net clinical benefit. The DeLong test showed that the AUC of the combined model was significantly higher than that of the clinical model (P = 0.032), but did not differ significantly from that of the RDL model (P = 0.161).

**Conclusion:**

The multimodal MRI radiomics-clinical fusion model accurately predicts IVGC treatment response in TED, offering a non-invasive tool for personalized therapy planning.

## 1 Introduction

Thyroid Eye Disease (TED), also known as thyroid-associated ophthalmopathy (TAO), is an organ-specific autoimmune disorder most commonly associated with Graves’ hyperthyroidism, predominantly involving the orbital soft tissues (1, 2). Key clinical manifestations include orbital inflammation, soft tissue infiltration, extraocular muscle hypertrophy, and proptosis, which may progress to compressive optic neuropathy or even blindness in severe cases (3, 4). Current treatment strategies for TED are largely guided by disease activity and severity (5, 6). Intravenous glucocorticoid (IVGC) pulse therapy remains the first-line treatment for moderate-to-severe active TED patients with a Clinical Activity Score (CAS) ≥ 3 (4, 7, 8). However, treatment efficacy varies considerably among individuals, with approximately 42% of patients showing primary resistance or intolerance to IVGC (9–12). Although CAS is widely used to assess inflammatory status, its subjective nature limits its accuracy in predicting individual therapeutic outcomes (13). Multimodal predictors can complement CAS: syMRI-derived T1/T2 relaxation times independently correlate with TAO activity and improve active–inactive discrimination when combined with a clinical indicator (AUC ≈ 0.88) (14), while adding ocular signs to clinical variables enhances IVGC response prediction (AUC 0.821 vs. 0.701), with conjunctival edema highlighted by SHAP as a key contributor (15).Thus, there is a compelling need to develop integrated predictive models incorporating multimodal biomarkers to optimize IVGC therapy and improve clinical decision-making.

Magnetic Resonance Imaging (MRI) has emerged as an indispensable tool for evaluating TED due to its excellent soft tissue contrast, allowing detailed visualization of morphological changes in orbital adipose tissue, extraocular muscles (EOMs), lacrimal glands, and the optic nerve (16–19). It enables detailed visualization of morphological alterations in orbital adipose tissue, extraocular muscles, lacrimal glands, and the optic nerve. Quantitative MRI parameters, such as T2 relaxation time and fat fraction (FF), further allow non-invasive assessment of tissue microstructure (4, 20). Advanced techniques including Dixon and T2-weighted imaging (T2WI) provide objective and quantitative measures that contribute to the evaluation of disease severity and progression (21). Previous efforts to enhance prediction accuracy have combined TSHR-Ab levels with extraocular muscle motility restriction, achieving an AUC of 0.861 (22), though such models did not incorporate radiomic data. Another study reported that a model integrating serum cholesterol and the minimum signal intensity ratio of extraocular muscles (EOM-SIRmin) had moderate predictive value (AUC = 0.834) (23), yet it was not validated using deep learning approaches.

Recent advances in artificial intelligence have accelerated the application of deep learning in TED. Convolutional neural networks (CNNs) applied to orbital CT images have demonstrated diagnostic-level accuracy in TAO screening (AUC = 0.919) (24). Deep transfer learning (DTL), which leverages pre-trained models on large-scale datasets followed by fine-tuning on limited medical imaging data, has effectively addressed challenges related to small sample sizes and facilitated multimodal data fusion (25, 26). This approach enables automated extraction of discriminative imaging features and supports knowledge transfer from general visual tasks to domain-specific applications, thereby improving model generalizability and robustness. Moreover, deep learning models are capable of learning hierarchical feature representations that more comprehensively capture complex disease phenotypes. For example, a nomogram combining T2 mapping-derived radiomics and clinical variables exhibited outstanding predictive performance (AUC = 0.952) (27), underscoring the promise of multimodal integration. Another investigation revealed that a radiomic signature (Rad-score) based on T2WI significantly outperformed the conventional EOM-SIRmin in predicting IVGC response (AUC = 0.968 vs. 0.745, p = 0.003) (20). These findings suggest complementary strengths between deep learning-derived and conventional radiomic features (28, 29), supporting their combined use to boost predictive accuracy.

Despite these developments, no study has yet integrated MRI-based radiomics with clinical predictors to characterize interpatient heterogeneity in response to IVGC pulse therapy in TED. To bridge this gap, we aimed to develop a deep learning-based image-clinical fusion model using multi-center MRI and clinical data. This model is designed to holistically evaluate morphological and pathophysiological traits in TED patients and assess whether a multidimensional predictor can enhance the accuracy of IVGC response prediction. Ultimately, this approach may help guide personalized treatment strategies, minimize ineffective steroid exposure, and improve patient prognosis.

## 2 Materials and methods

### 2.1 General clinical data

This retrospective multicenter study enrolled 108 patients with a confirmed diagnosis of TED from the Second Hospital of Lanzhou University and Lanzhou First People’s Hospital between January 2020 and December 2024. Based on their response to intravenous glucocorticoid (IVGC) therapy assessed at 12 weeks post-treatment completion, patients were classified into responsive (n=78) and non-responsive (n=30) groups. The cohort was randomly partitioned into training and test sets using an 8:2 ratio. All patients fulfilled the Bartley diagnostic criteria (1), presented with a Clinical Activity Score (CAS) ≥3, and completed a standardized methylprednisolone pulse therapy regimen.

This study was conducted in accordance with the Declaration of Helsinki and received approval from all participating institutional review boards (Approval No.: 2025A-918), with waiver of informed consent granted for retrospective data analysis. All patient data were de-identified prior to analysis.

Inclusion criteria were: (1) Age ≥ 18 years; (2) Bilateral eye involvement; (3) Absence of complex systemic diseases or other orbital conditions; (4) No prior glucocorticoid or ocular-related treatments; (5) Complete clinical data and MRI images meeting diagnostic quality requirements; (6) Completion of the IVGC regimen according to the 2021 European Group on Graves’ Orbitopathy (EUGOGO) guidelines (total dose 4.5 g over 12 weeks).

The Clinical Activity Score (CAS) comprised 7 items: spontaneous retrobulbar pain, pain on eye movement, eyelid erythema, eyelid edema, conjunctival redness, chemosis, and swelling of the caruncle, each scoring 1 point. CAS ≥ 3 indicated active disease, while CAS < 3 indicated inactive disease. Diagnostic criteria for moderate-to-severe TED included: (1) Eyelid retraction (≥2 mm); (2) Moderate or severe soft tissue involvement; (3) Proptosis ≥3 mm; (4) Inconstant or constant diplopia. Each eye underwent the following ophthalmic assessments before and after IVGC treatment: (1) CAS score; (2) Exophthalmometry; (3) Intraocular pressure (IOP) measurement; (4) Diplopia score.

### 2.2 Clinical evaluation and treatment protocol

Standardized ophthalmic assessments were performed at baseline and at 12 weeks after completion of IVGC treatment. Assessments included CAS evaluation, proptosis measurement, intraocular pressure measurement, and diplopia evaluation.

All patients received intravenous methylprednisolone according to the EUGOGO-recommended regimen: 500 mg weekly for 6 weeks followed by 250 mg weekly for an additional 6 weeks (total dose: 4.5 g over 12 weeks). The 24-week follow-up protocol included baseline documentation, 12-week therapeutic response evaluation, and subsequent 12-week outcome monitoring.

Treatment efficacy was determined using both patient-reported outcomes (PROs) via the GO-QOL questionnaire and clinician-reported outcomes (CROs). Objective response criteria included: (1) ≥2 mm reduction in eyelid retraction; (2) ≥1-point decrease in the five non-pain CAS components; (3) ≥2 mm reduction in proptosis; and/or (4) ≥8-degree improvement in ocular motility. Treatment success was defined as meeting ≥2 objective criteria in the studied eye without deterioration in the contralateral eye.

### 2.3 MRI acquisition protocol

Orbital MRI was performed using a Philips 3.0T scanner with a 32-channel head coil. Standard imaging parameters included: fast spin-echo T1WI(FOV 180×180 mm, TR/TE 570/9 ms, slice thickness/gap 3/0.3 mm, matrix 256×256, NEX = 2) and T2WI-FS (FOV 180×180 mm, TR/TE 2400/90 ms, slice thickness/gap 3/0.3 mm, matrix 256×256, NEX = 2).

### 2.4 Radiomics analysis

#### 2.4.1 Image segmentation and preprocessing

**Figure 1:**
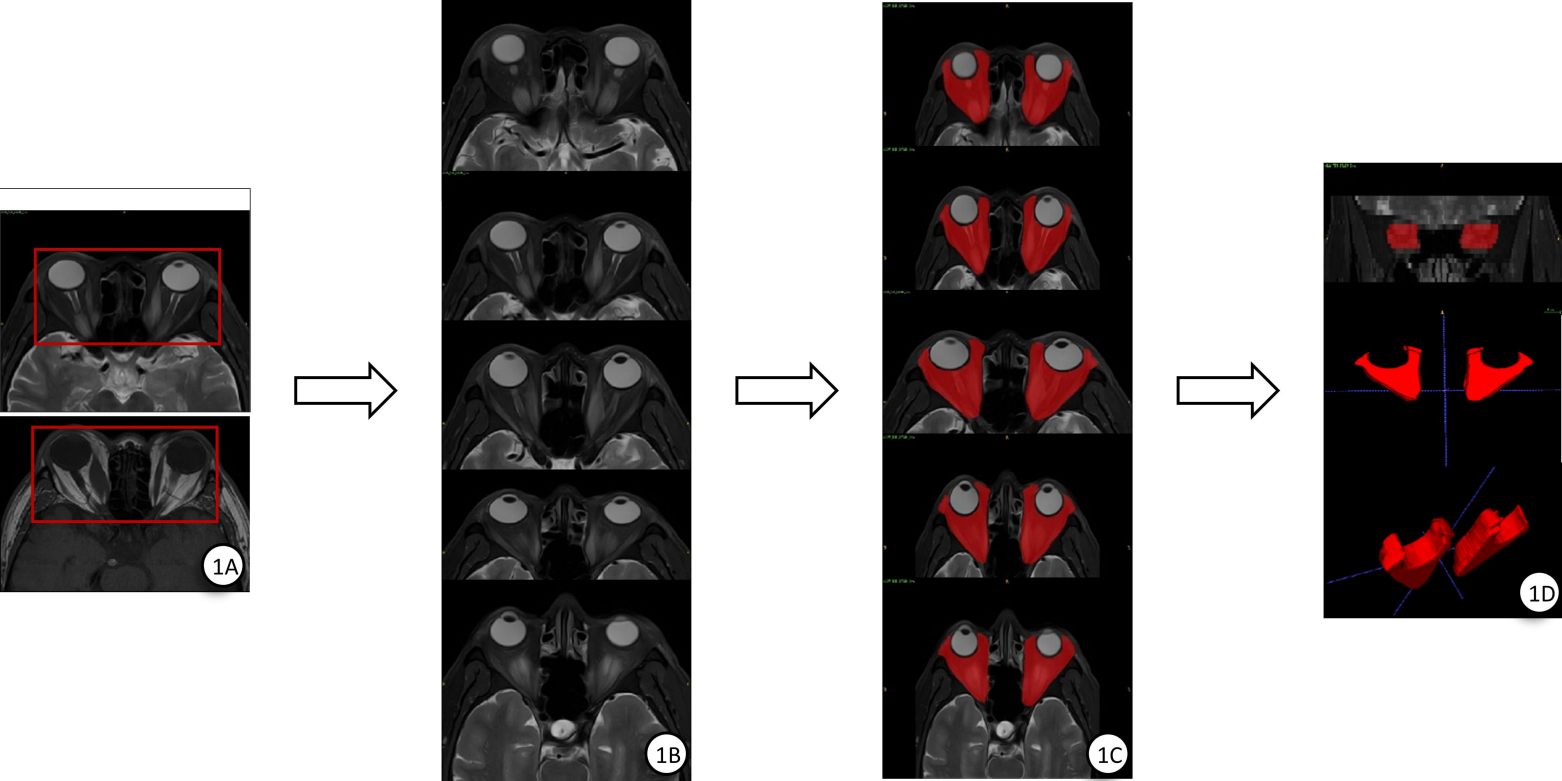
Schematic diagram illustrating the slice-by-slice delineation process for constructing a three-dimensional volume of interest (VOI) along the optic nerve level on T2-weighted imaging (T2WI). **(A)** Representative original axial T1WI and T2WI-FS images. **(B, C)** Sequential five image slices centered on the optic nerve level, demonstrating the manually delineated two-dimensional regions of interest (ROIs) highlighted in red. **(D)** The final three-dimensional orbital VOI rendered by stacking the series of two-dimensional ROIs.

All patients underwent MRI examination before treatment. A radiologist with over five years of experience in ophthalmic imaging manually delineated the Regions of Interest (ROIs) on axial T1WI and T2WI-FS sequences (25), using ITK-SNAP 4.0(http://www.itksnap.org). Delineation was performed slice-by-slice, centered on the optic nerve level and covering five consecutive slices superiorly and inferiorly (30), as illustrated in **Figure 1**. The corresponding two-dimensional ROIs were then stacked to reconstruct three-dimensional volumes of interest (VOIs) encompassing the lesion areas. Subsequently, all images underwent N4 bias-field correction and intensity normalization (31), and were resampled to a standardized voxel resolution of 1mm ×1mm ×1mm (32).

#### 2.4.2 Feature extraction and quality control

Radiomics features were extracted using the open-source Pyradiomics package on the Python platform(https://github.com/AIM-Harvard/pyradiomics) (33, 34). To verify inter-observer consistency of feature extraction, 30 randomly selected cases from the training set were re-contoured by a second radiologist with ten years of ophthalmic imaging experience, and features were re-extracted. Intraclass correlation coefficient (ICC) was used to assess consistency; only features with ICC > 0.80 were retained to ensure reliability. Pixel intensities were normalized using Z-score normalization to stabilize model training, resulting in a distribution with zero mean and unit standard deviation. A ResNet-50 convolutional neural network pre-trained on the ImageNet dataset served as the base model for deep learning feature extraction (35).

#### 2.4.3 Feature selection and integration

First, independent samples t-tests (P < 0.05) were applied to radiomics features to select those showing significant differences (36). Subsequently, Pearson correlation analysis was performed, and features with correlation coefficients > 0.9 were removed to eliminate high redundancy. Next, the LASSO algorithm was employed for feature selection. The optimal penalty coefficient λ was determined via ten-fold cross-validation, and the subset of features corresponding to non-zero coefficients was retained (34, 35, 37), resulting in an independent and stable feature set. Selected features were standardized using Z-score normalization. For the high-dimensional DTL features, Principal Component Analysis (PCA) was applied for dimensionality reduction (38), retaining principal informative components. Standardized radiomics features and dimensionality-reduced DTL features were then fused via early fusion to form a comprehensive feature set. LASSO was applied again to this fused set to select features with non-zero coefficients, yielding a final optimal fused feature subset of 32 dimensions.

#### 2.4.4 Model construction and validation

After feature fusion, a Multinomial Logistic Regression (MLR) classifier (39, 40) was used. Ten-fold cross-validation combined with a grid search algorithm was employed for hyperparameter tuning, identifying the optimal model configuration by iterating through predefined parameter combinations (5). Model performance was evaluated using metrics including accuracy, specificity, sensitivity, and the Area Under the receiver operating characteristic Curve (AUC).

### 2.5 Statistical analysis

All analyses were conducted using SPSS 25.0, Python 3.7, and R 4.0.2. Continuous variables are presented as mean ± standard deviation or median ± interquartile range for normally and non-normally distributed data, respectively. Model comparisons employed DeLong’s test for AUC differences, while clinical utility was evaluated through decision curve analysis. The final combined model incorporating radiomic, deep learning, and clinical features was visualized as a nomogram. Statistical significance was set at P<0.05.

## 3 Results

### 3.1 Comparison of baseline clinical characteristics between training and test sets

This study included 108 patients with TED, with ages ranging from 25 to 86 years and a mean age of 49.87 ± 13.54 years. Based on treatment response, patients were categorized into responsive (78 patients) and non-responsive (30 patients) groups. To ensure validity and reliability, all cases were randomly divided into training and test sets in an 8:2 ratio. The training set comprised 86 patients, and the test set comprised 22 patients. Detailed clinical data, including but not limited to age, disease duration, CAS, and proptosis, were recorded for all patients. Statistical summaries of the distribution for all clinical features are detailed in **Table 1**.

**Table 1:** Clinical baseline characteristics of TED patients in the training and test sets.

Clinical characteristic	Total sample (n=108)	Training set (n=86)	Test set (n=22)	p-value
Age (years)	49.87 ± 13.54	50.12 ± 14.00	48.91 ± 11.80	0.680
Disease Duration (months)	20.52 ± 51.12	22.85 ± 56.94	11.41 ± 9.27	0.695
CAS Score	4.14 ± 1.21	4.15 ± 1.15	4.09 ± 1.44	0.552
OD(mm)	20.72 ± 3.36	20.80 ± 3.45	20.41 ± 3.03	0.632
OS(mm)	20.41 ± 3.91	20.16 ± 3.96	21.37 ± 3.63	0.524
Lateral Rectus (Right, mm)	4.65 ± 1.50	4.64 ± 1.53	4.69 ± 1.39	0.792
Medial Rectus (Right, mm)	5.06 ± 2.94	5.05 ± 2.90	4.84 ± 3.17	0.757
Inferior Rectus (Right, mm)	5.54 ± 2.26	5.66 ± 2.28	5.08 ± 2.17	0.179
Lateral Rectus (Left, mm)	4.49 ± 1.25	4.52 ± 1.29	4.37 ± 1.08	0.660
Medial Rectus (Left, mm)	5.36 ± 1.95	5.37 ± 1.96	5.30 ± 1.92	0.834
Superior Rectus (Left, mm)	5.70 ± 2.42	5.77 ± 2.44	5.41 ± 2.39	0.393
Inferior Rectus (Left, mm)	6.30 ± 2.36	6.31 ± 2.26	6.25 ± 2.76	0.397
Lacrimal Gland Proptosis (Right, mm)	12.68 ± 2.61	12.80 ± 2.59	12.19 ± 2.69	0.332
Lacrimal Gland Proptosis (Left, mm)	12.38 ± 3.03	12.32 ± 3.14	12.63 ± 2.62	0.668
Lacrimal Gland Diameter (Right, mm)	3.95 ± 1.37	4.01 ± 1.42	3.72 ± 1.18	0.680
Lacrimal Gland Diameter (Left, mm)	3.95 ± 1.14	3.92 ± 1.12	4.08 ± 1.24	0.529
Optic Nerve Diameter (Right, mm)	4.31 ± 0.67	4.35 ± 0.69	4.14 ± 0.59	0.321
Optic Nerve Diameter (Left, mm)	4.35 ± 0.70	4.36 ± 0.73	4.32 ± 0.59	0.817
Gender				0.655
Male	61(56.48)	50(58.14)	11(50.00)	
Female	47(43.52)	36(41.86)	11(50.00)	

CAS, Clinical Activity Score; OD, Exophthalmos of the right eye; OS, Exophthalmos of the left eye.

Univariate logistic regression analysis was performed to identify independent risk factors affecting treatment efficacy. The results indicated that disease duration and CAS (P < 0.05) were identified as clinical independent risk factors associated with TED treatment response.

### 3.2 Construction of the radiomics fusion model

**Figure 2:**
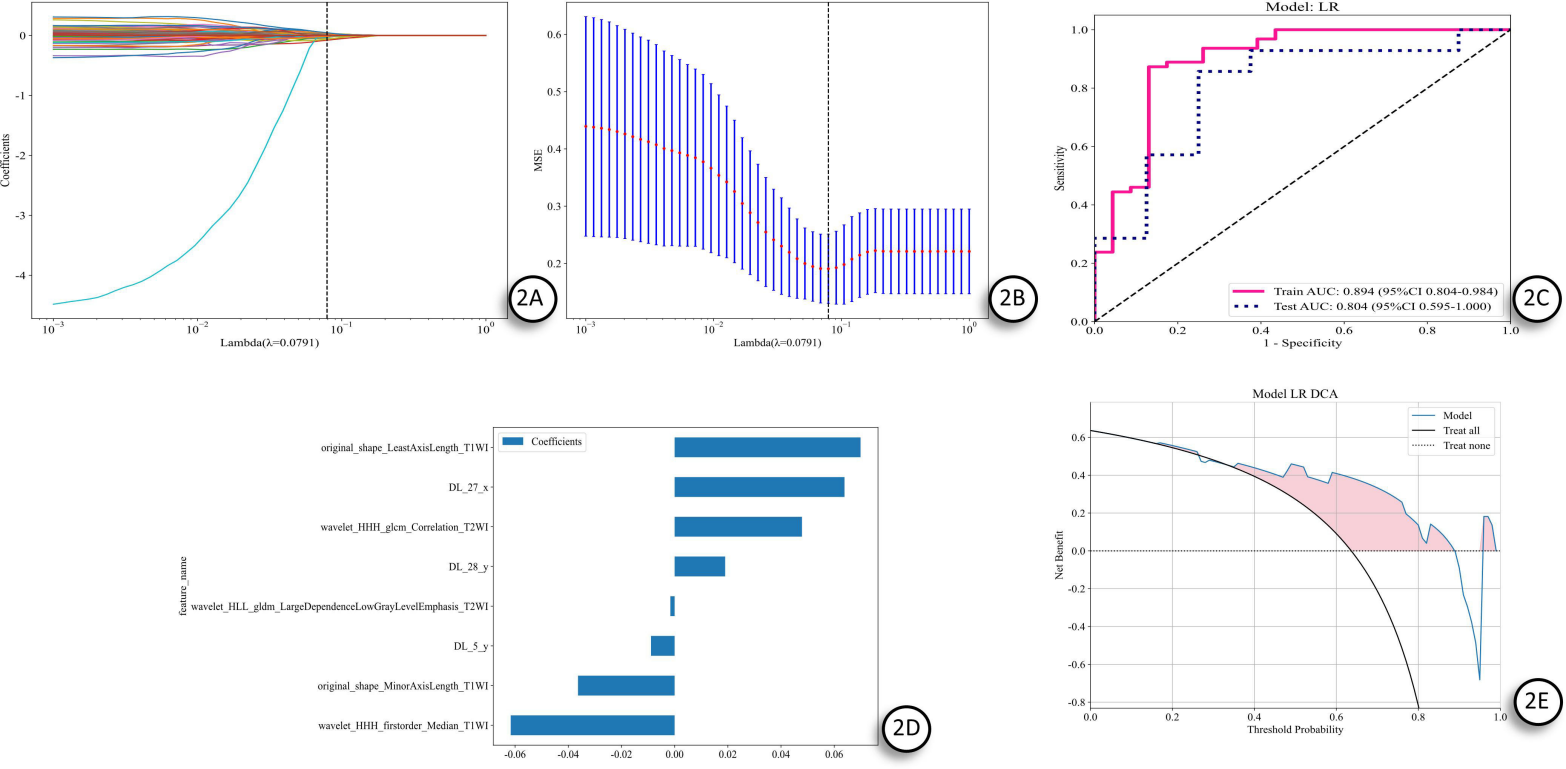
LASSO–RDL Model Development and Evaluation. **(A)** LASSO path and optimal λ≈0.0791; **(B)** Cross-validation MSE–λ curve; **(C)** ROC: Training AUC = 0.894 (95% CI 0.804–0.984), Testing AUC = 0.804 (95% CI 0.595–1.000); **(D)** Selected features and standardized coefficients; **(E)** DCA shows positive net benefit of the model across common threshold ranges. LASSO, Least Absolute Shrinkage and Selection Operator; LR, Logistic Regression; ROC, Receiver Operating Characteristic; AUC, Area Under the Curve; CI, Confidence Interval; MSE, Mean Squared Error; DCA, Decision Curve Analysis.

Radiomics features and deep learning (DL) features were extracted from T1-weighted imaging (T1WI) and T2-weighted fat-saturated imaging (T2WI-FS) scans. To ensure feature reliability, an intraclass correlation coefficient (ICC) analysis was conducted, which resulted in the retention of 2394 radiomics features and 2458 DL features. Feature selection was performed using LASSO regression, with the coefficient path plot (**Figure 2A**) illustrating that as the regularization parameter (λ) increased, most feature coefficients were driven to zero. Ten-fold cross-validation was employed to identify the optimal λ value, which was determined to be 0.0791 (**Figure 2B**). At this threshold, a total of 3 T1WI radiomics features, 2 T2WI radiomics features, and 3 DTL features were selected, forming a final feature set of 8 features, creating a radiomics-deep learning fusion feature set (**Figure 2D**).

The radiomics-deep learning (RDL) model, constructed using this feature set, achieved an area under the curve (AUC) of 0.894 in the training set and 0.804 in the test set (**Figure 2C**). Decision curve analysis (DCA) (**Figure 2E**) further confirmed that the RDL model exhibited excellent predictive performance and clinical decision-making value in predicting TED patient response.

### 3.3 Development and validation of the combined predictive model

To further enhance predictive accuracy, we combined two independent clinical risk factors—disease duration and Clinical Activity Score (CAS)—with features from the radiomics-deep learning (RDL) model to construct a combined predictive model. The combined model demonstrated AUCs of 0.916 (95% CI 0.837-0.994) in the training set and 0.862 (95% CI 0.702-1.000) in the test set. These values were higher than those achieved by the clinical model alone (training set AUC: 0.834, 95% CI 0.741-0.926; test set AUC: 0.728, 95% CI 0.497-0.958) and the RDL model alone (training set AUC: 0.894, 95% CI 0.804-0.984; test set AUC: 0.804, 95% CI 0.595-1.000).Detailed data are presented in **Table 2**. Additionally, the combined model exhibited the highest specificity in the test set (0.875), while maintaining high accuracy (0.818) and sensitivity (0.786).

**Table 2:** Predictive of models for the responsiveness of TED patients to IVGC treatment.

Models	Accuracy/%	AUC	95% CI	Sensitivity/%	Specificity/%
Training					
Clinic	0.756	0.834	0.7412-.926	0.698	0.913
RDL	0.872	0.894	0.804 -0.984	0.873	0.870
combined	0.895	0.916	0.837 - 0.994	0.937	0.783
Test					
Clinic	0.727	0.728	0.497 - 0.958	0.714	0.750
RDL	0.818	0.804	0.595 - 1.000	0.857	0.750
combined	0.818	0.862	0.702- 1.000	0.786	0.875

Clinic, clinical prediction model; RDL, radiomics and deep learning model; Combined, RDL model with clinical prediction model—disease duration and Clinical Activity Score (CAS).

### 3.4 Model calibration, clinical utility, and development of an individualized predictive tool

**Figure 3:**
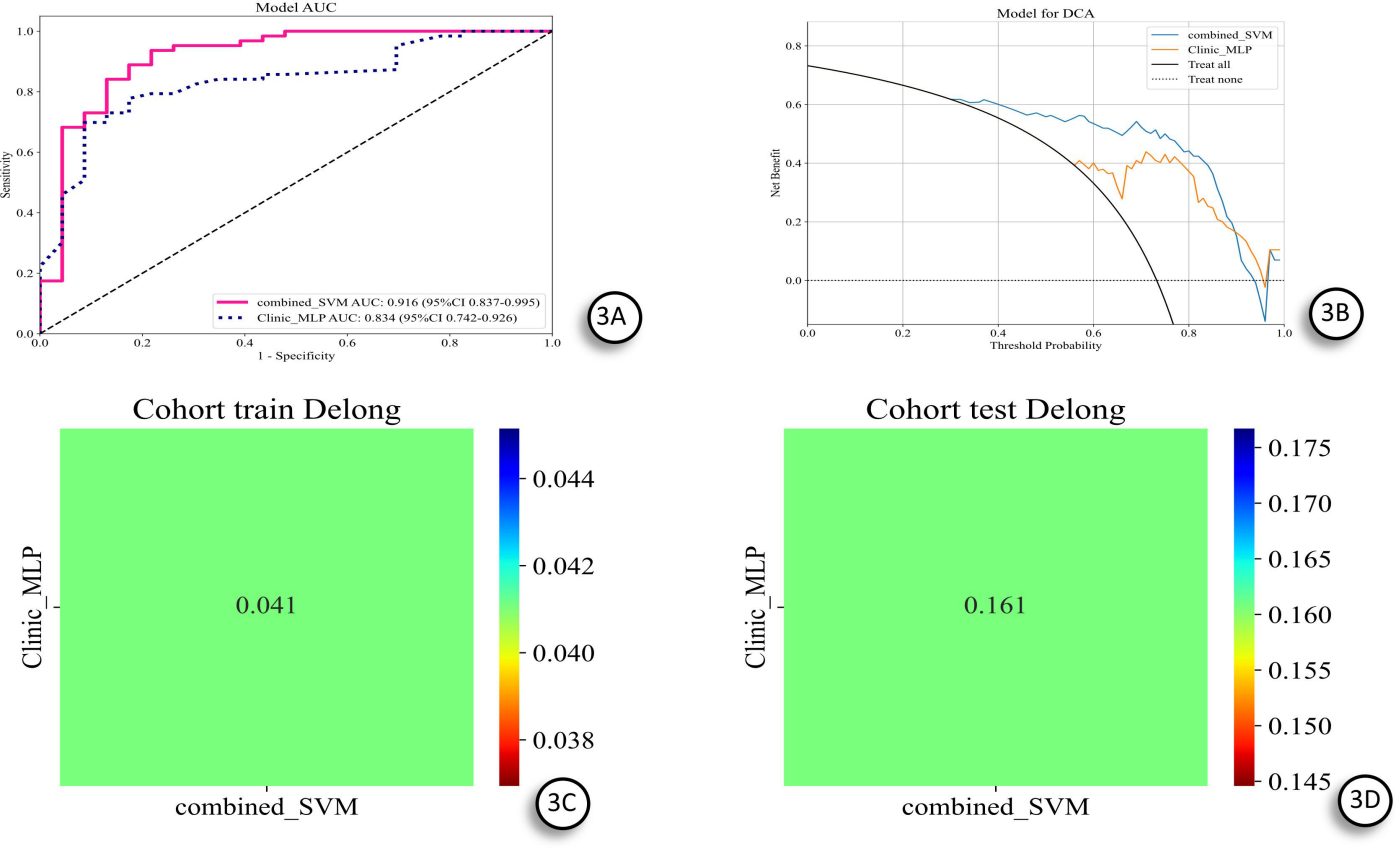
Presents the ROC curves and decision curve analysis (DCA) for the combined model and the Clinic model in predicting the response to IVGC treatment in patients with Thyroid Eye Disease. **(A)** ROC curve analysis; AUC: area under the curve; combined: combined model; Clinic: clinical model. **(B)** DCA curve analysis. **(C)** Delong test comparison between Clinic_MLP and combined_SVM models in the training cohort. **(D)** Delong test comparison between Clinic_MLP and combined_SVM models in the testing cohort.

The combined model demonstrated excellent predictive performance in both the training and test sets. To quantify the statistical significance of the AUC differences between models, we performed pairwise comparisons using the DeLong test. In the training set, the combined model’s AUC (0.916) was notably higher than both the clinical model (0.834) and the RDL model (0.894) (**Figures 3A, B**). However, the DeLong test indicated that the AUC difference between the combined and clinical models did not reach statistical significance (P = 0.041), and the difference with the RDL model also did not reach significance (P > 0.05) (**Figures 3C**). In the independent test set, the combined model still showed the highest AUC (0.862). The DeLong test revealed that the AUC difference between the combined and clinical models was statistically significant (P = 0.032), but the difference with the RDL model (AUC = 0.804) was not statistically significant (P = 0.161) (**Figure 3D**). This result suggests that, although the combined model exhibited the best performance trend in the test set, its incremental improvement over the pure imaging fusion model (RDL) cannot be ruled out as a result of chance, given the current sample size.

**Figure 4:**
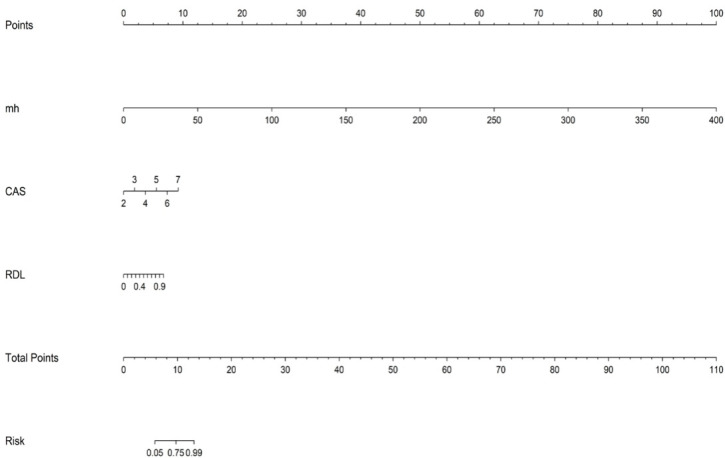
Nomogram for the combined model. The higher the total score corresponding to different indicators, the greater the predicted effectiveness of IVGC treatment response in Thyroid Eye Disease patients. Points, score; mh, disease duration; CAS, Clinical Activity Score; RDL, radiomics fusion model; Total Points, total score; Risk, risk value.

To translate the combined predictive model into an easy-to-use tool for clinical practice, we developed a visual nomogram based on the results of multivariate logistic regression (**Figure 4**). This nomogram integrates all the predictive factors from the model: disease duration (mh), Clinical Activity Score (CAS), and the radiomics-deep learning fusion score (RDL Score). By locating the patient’s specific indicators along the corresponding axes and summing the total score, clinicians can read the predicted risk probability on the “Risk” axis. This nomogram provides a straightforward and convenient tool for clinical decision-making, facilitating more personalized risk assessment.

## 4 Discussion

This study successfully developed and validated a multimodal fused nomogram model based on radiomics features, deep transfer learning (DTL) features, RDL fused features, and clinical features, systematically evaluating its clinical value for predicting TED patient responsiveness prior to IVGC therapy. The results demonstrated that this model exhibited excellent diagnostic performance in both the training set (AUC = 0.916, 95% CI 0.837–0.994) and the test set (AUC = 0.862, 95% CI 0.702–1.000), significantly outperforming single-modality models. This study not only provides a robust decision-making basis for formulating individualized treatment strategies for TED patients but also innovatively highlights the pivotal role of RDL-clinical fused features in enhancing predictive efficacy, suggesting their potential as a novel biomarker in TED diagnosis and treatment.

### 4.1 Radiomics research on IVGC treatment responsiveness in TED patients

With the rapid advancement of artificial intelligence technology, radiomics, as a crucial tool in translational medicine, enables in-depth analysis of lesion biological characteristics through high-throughput extraction and quantitative analysis of microscopic features from medical images (41). In TED diagnosis and treatment, traditional imaging assessment methods are limited by subjectivity and struggle to accurately predict IVGC treatment response. Several recent studies have made progress in this direction: Hu et al. established a non-invasive prediction model for glucocorticoid treatment response in TED patients by combining T2WI radiomics features with disease duration parameters (20); Wang et al. developed a novel prediction scheme capable of early identification of Graves’ ophthalmopathy (GO) patients unresponsive to intravenous corticosteroid pulse therapy (42); Zhang et al. achieved favorable IVGC treatment response prediction in TED patients using a WOR model (23); Park et al. demonstrated, via the XGBoost algorithm, that extraocular muscle restriction and thyrotropin receptor antibody levels significantly influence treatment response (22). More recently, whole-orbit radiomics, which integrates features across multiple orbital compartments, has outperformed single-region radiomics and semiquantitative imaging, highlighting the value of comprehensive orbital phenotyping for IVGC response prediction (43).

Centered on the optic nerve, we selected five axial levels spanning superior and inferior slices and encompassing multiple orbital soft-tissue regions (e.g., extraocular muscles, lacrimal gland, orbital fat, and the optic nerve). We then incorporated deep learning features extracted with a ResNet50 backbone to build an RDL model that fuses radiomic and deep learning representations. This RDL model outperformed both the radiomics-only model and the single-region radiomics model, highlighting the added value of multimodal fusion and multi-compartment sampling for capturing deeper imaging patterns.

### 4.2 Application value and efficacy analysis of the predictive model for IVGC treatment responsiveness in TED patients

This study, utilizing T1WI and T2WI-FS sequences for feature extraction, fused radiomics features and DTL features to construct an innovative RDL fusion model. The final selection included 3 T1WI radiomics features, 2 T2WI radiomics features, and 3 DTL features. Morphological features reflect the orbital anatomical structure, texture features characterize the heterogeneity of grayscale spatial distribution, while deep learning features capture higher-level imaging patterns through non-linear mapping. This multi-dimensional feature fusion strategy effectively compensates for the limited representational capacity of single modalities.

After further integrating the two clinical indicators—disease duration and CAS score—the combined model’s AUC increased to 0.916 in the training set and 0.862 in the test set, significantly superior to using the RDL model alone (P < 0.05). This performance improvement can be attributed to the following aspects: 1) Three-dimensional texture analysis more comprehensively captures the spatial heterogeneity of tissues (44); 2) RDL features supplement high-level semantic information not covered by traditional radiomics (45); 3) Clinical indicators enhance the model’s ability to assess disease activity (44). The visualization of this model via a nomogram significantly improves its clinical applicability and decision-support capability.

Notably, beyond confirming disease duration and CAS as key clinical predictors, the fusion model captured complementary imaging-derived information. The radiomics–DTL fused signature provided incremental discrimination compared with the clinical-only model, supporting the presence of imaging biomarkers associated with IVGC responsiveness.

This study validates the significant advantage of multimodal feature fusion in predicting TED treatment response. Future work could involve multi-center validation to assess model robustness and integrate biomarkers from more dimensions, such as genomics, to build a more precise individualized prediction system.

### 4.3 Clinical visualization, calibration, and risk stratification

To translate the model into clinical practice, we visualized the predictive tool as an intuitive nomogram, which integrates disease duration, CAS, and the RDL score. This enables clinicians to make individualized risk predictions regarding IVGC treatment response in a simple, interpretable format. Calibration curve analysis showed that the model provided reliable risk estimations, with slopes near 1 and low Brier scores, both in the training and test sets. In contrast, the clinical-only model exhibited poor calibration, especially in the test set, underscoring its limited utility for personalized risk prediction.

This clinical visualization enables effective risk stratification. High-risk patients identified by the model can be considered for alternative treatments or early combination therapies, thus preventing unnecessary IVGC treatment and improving clinical outcomes. Decision curve analysis (DCA) further validated the model’s clinical utility by demonstrating positive net benefit across a wide range of decision thresholds, particularly between 0.4 and 0.5, which highlights the model’s value in supporting clinical decision-making.

### 4.4 Limitations of this study

This study constructed a multimodal predictive model integrating radiomics, deep transfer learning features, and clinical indicators, capable of providing an individualized, non-invasive predictive tool for IVGC treatment responsiveness in TED patients, thereby aiding the optimization of clinical treatment decisions. However, several limitations exist: First, although data from two centers were included and a retrospective analysis was conducted, selection bias may still be present, and the lack of validation from more external centers limits generalizability; subsequent plans include expanding the sample sources to enhance model reliability. Second, although deep learning features show promising predictive potential, the limited sample size and the challenge of feature interpretability remain; future studies need larger samples to optimize model fitting and efficacy. Finally, the current ROI delineation was based solely on axial T1WI and T2WI sequences; future work will incorporate more sequences to improve information completeness and model generalizability. In addition, although we leveraged transfer learning and regularization strategies to mitigate overfitting in a limited-sample setting, the cohort size remains modest for deep learning. Future work should include larger multi-center cohorts and independent external validation to further confirm generalizability.

## Data Availability

The raw data supporting the conclusions of this article will be made available by the authors, without undue reservation.
